# Relationship between Undescended Testis Position and Prevalence of Testicular Appendices, Epididymal Anomalies, and Patency of Processus Vaginalis

**DOI:** 10.1155/2017/5926370

**Published:** 2017-12-28

**Authors:** Luciano A. Favorito, Helce Riberio Julio-Junior, Francisco J. Sampaio

**Affiliations:** Urogenital Research Unit, State University of Rio de Janeiro, Rio de Janeiro, RJ, Brazil

## Abstract

**Objectives:**

To assess the incidence of testicular appendices (Tas), epididymal anomalies (EAs), and processus vaginalis (PV) patency in patients with undescended testis (UT) according to testicular position and to compare them with human fetuses.

**Methods:**

We studied 85 patients (108 testes) with cryptorchidism and compared the features with those of 15 fetuses (30 testes) with scrotal testes. We analyzed the relationships among the testis and epididymis, patency of PV, and the presence of TAs. We used the Chi-square test for statistical analysis (*p* < 0.05).

**Results:**

In 108 UT, 72 (66.66%) had PV patent, 67 (62.03%) had TAs, and 39 (36.12%) had EAs. Of the 108 UT, 14 were abdominal (12.96%; 14 had PV patency, 9 TAs, and 7 EAs); 81 were inguinal (75%; 52 had PV patency, 45 TAs, and 31 EAs), and 13 were suprascrotal (12.03%; 6 had PV patency, 13 TAs, and 1 EAs). The patency of PV was more frequently associated with EAs (*p* = 0.00364). The EAs had a higher prevalence in UT compared with fetuses (*p* = 0.0005).

**Conclusions:**

Undescended testis has a higher risk of anatomical anomalies and the testes situated in abdomen and inguinal canal have a higher risk of presenting patency of PV and EAs.

## 1. Introduction

During the human fetal period, the testes migrate from the abdomen to the scrotum, traversing the abdominal wall and the inguinal canal between the 15th and the 28th week postconception [1, 2]. Cryptorchidism is one of the most common congenital anomalies among males, with a rate between 2 and 5% of full-term births, a rate that can reach 30% in premature babies [3, 4].

Cryptorchidism can be associated with various anatomical anomalies, but epididymal anomalies and patency of the vaginal process are among the most frequent [3, 5, 6]. Epididymal anomalies are associated with cryptorchidism with highly variable incidence reported in the literature: from 36 to 79% [7, 8]. The occurrence of inguinal hernias associated with cryptorchidism is due to the persistence of the vaginal process [9, 10]. The vaginal process (PV) is a conduit that extends from the peritoneum to the scrotum and is covered by a coelomic epithelium. This conduit is usually obliterated after the end of the testicular migration [9, 10]. In cases where the vaginal process does not close, the child may develop inguinal hernia or communicating hydrocele.

Testicular and epididymal appendices have been considered congenital anomalies [9]; however some studies report that these structures are present in most normal individuals [11]. The functions of testicular appendages are controversial: they can control the amount of serous fluid in the vaginal tunica space [12].

Studies of epididymal anomalies and their relation to the patency of the vaginal process as well as the analysis of the embryology and structure of the testicular and epididymal appendages are frequent [11, 13, 14]. The analysis of the correlation between the position of the cryptorchidic testis and the presence of abnormalities of the epididymis, patency of the vaginal process, and testicular appendages are rare. To the best of our knowledge, no study has been published regarding these parameters using as a control group human fetuses with testes that completed their migration.

The aim of this paper is to assess the incidence of testicular appendices (Tas), epididymal anomalies (EAs), and processus vaginalis (PV) patency in patients with undescended testis (UT) according to testicular position and compare these aspects with human fetuses having testes situated in scrotum.

## 2. Material and Methods

This study was approved and was carried out in accordance with the ethical standards of the hospital's institutional committee on human experimentation.

From January 2011 to January 2017, we studied 85 patients with cryptorchidism (108 testicles). Fifteen male human fetuses with the scrotal testes were also studied. Fetuses were of gestational age between 30 and 35 weeks postconception (WPC) and their cause was prematurity or perinatal asphyxia.

All patients underwent orchidopexy through incision in the inguinal region and the testicles were divided into three groups according to their position: (A) abdominal: testicles located above the inner inguinal ring; (B) inguinal: testicles located between the inner and outer inguinal rings; and (C) suprascrotal: testicles located below the outer inguinal ring. During surgery, three parameters were analyzed: (A) persistence of PV; (B) relationship between testis and epididymis; and (C) presence of testicular appendages.

To analyze the relations between the testis and epididymis in surgical patients and fetuses, we used a previous classification [15, 16]: Type I: epididymis attached to the testis at the head and tail; Type II: epididymis totally attached to the testis; Type III: epididymis attached to the testis only at the head; Type IV: epididymis attached to the testis only at the tail; Type V: no visible connection between the testis and epididymis; and Type VI: epididymal atresia. Type I and II relationships are considered normal, while the other types are considered to be epididymal anomalies (EAs) [15]. To analyze the structure of the PV, we considered two situations: (a) complete obliteration of the PV between the internal inguinal ring and the upper pole of the testis and (b) complete patency of the PV.

**Figure 1:**
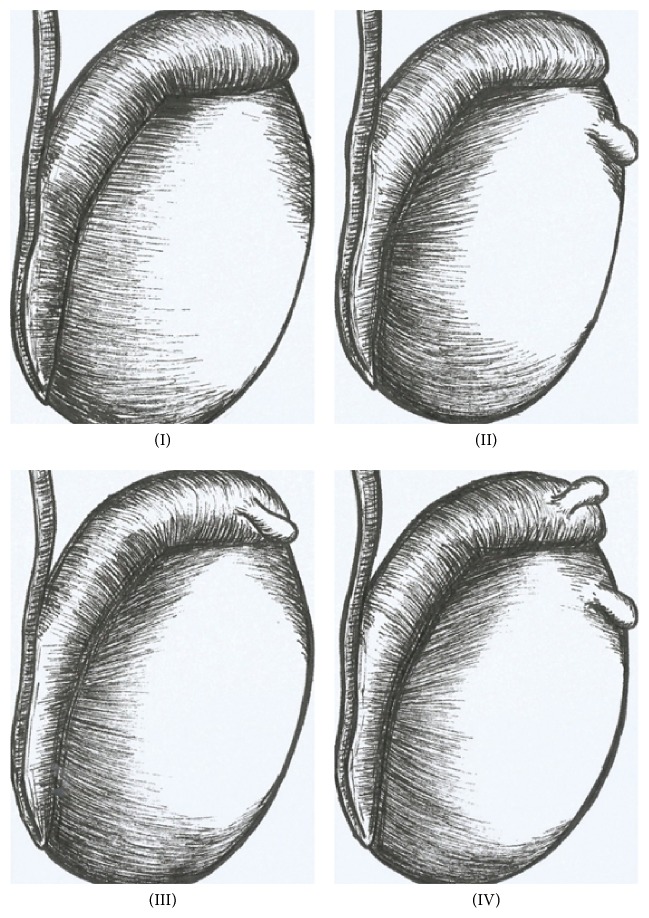
Schematic drawing showing the types of possible dispositions of the paratesticular appendages found during surgical intervention: (I) absence of testicular and epididymis appendages; (II) presence of testicular appendix; (III) presence of epididymal appendage; and (IV) presence of the testicular appendix and epididymis.

In relation to the testicular appendices, we analyzed the following situations: (I) absence of testicular and epididymal appendages, (II) presence of a testicular appendix, (III) presence of appendix of the epididymis, and (IV) presence of testicular appendix and epididymis (Figure 1).

The urogenital tract of the 15 fetuses studied was anatomically well preserved. To estimate the gestational age, the measurement of the length of the largest foot [17, 18] was used. We also measured the weight, total length and the vertex-coccyx length of the fetuses. All measurements were done by the same investigator. After the measurements, the fetuses were carefully dissected with the aid of a stereoscopic lens with 16/25x magnification.

We used the Chi-square test for contingency analysis of the populations under study (*p* < 0.05), calculated by GraphPad.

## 3. Results

The fetuses presented age between 30 and 35 WPC (mean = 31.66), weight between 1195 and 2860 g (mean = 1835.53), and VC length between 27 and 34 cm (mean = 30.67). PV was found in 7 cases (23.34%). We observed EAs (Type III) in only 1 case (3.44%). In 18 cases (60%) we observed the presence of a testicular appendix, while in 7 cases (23.33%) no appendices were found. In 3 cases (10%) we observed the presence of a testicular appendix and epididymis and in 2 cases (6.6%) we found a single epididymal appendix.

The 85 patients were aged between 1 and 10 years (mean = 5.16). Of the 108 testes, 14 were abdominal (12.96%); 81 inguinal (75%); and 13 suprascrotal (12.03%). The correlations between the position of the cryptorchid testicles, the presence of ATs, the patency of the PV, and the EAs can be seen in Table 1.

PV was found in 72 cases (66.66%) of cryptorchidism and in 7 cases (23.34%) in the fetuses (*p* < 0.0001). All abdominal testicles had patent PV, and this patency is significant in relation to fetuses (*p* < 0.001), the inguinal testicles (*p* = 0.0072), and also the suprascrotal testes (*p* = 0.0014). We also observed that the patency of PV in the testes located in the canal was higher than the patency in the fetuses (*p* = 0.0001). We did not observe any difference in the patency of the PV between the suprascrotal testis and the fetus (*p* = 0.1345) and between the inguinal and suprascrotal testes (*p* = 0.2141).

**Figure 2:**
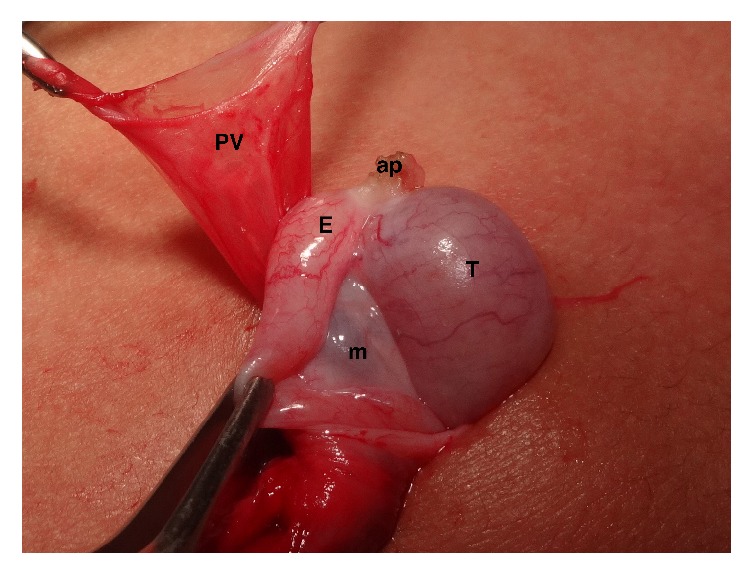
A 4-year-old patient with suprascrotal testis presented anatomical relationship between the testis and the epididymis Type I, epididymis attached to the testis at the head and tail. Note the presence of a testicular appendix (ap) and patency of the processus vaginalis (PV). m = mesorchium, T = testis, and E = epididymis.

EAs (Figure 2) were found in 39 cases of cryptorchidism (36.11%) and in only one case in the fetuses (*p* = 0.0005). We observed AEs in 7 abdominal (50%), 31 inguinal (38.27%), and 1 suprascrotal testes (7.69%). There was a difference between the presence of EAs in the abdominal (*p* = 0.0002) and inguinal (*p* = 0.0003) testicles compared to the fetuses and between the abdominal and suprascrotal testes (*p* = 0.0161). There was no significant difference in the occurrence of EAs between the suprascrotal testes and fetuses (*p* = 0.5330); between the abdominal and inguinal testes (*p* = 0.4082); and between the inguinal and suprascrotal testes (*p* = 0.0308).

Of the 72 testicles with patent PV, 27 (37.5%) had EAs, and in the 36 testicles that had occluded PV, 12 (33.33%) had EAs (*p* = 0.67). In 40 cases of AEs (39 in cryptorchidism and 1 in fetuses), the PV was patent in 29 (72.5%). On the other hand, in the 96 cases of normal anatomy of the epididymis (69 cryptorchidism and 29 in the fetuses), the PV was patent in 51 (53.12%), which was significant (*p* = 0.0364).

There were no differences (*p* = 0.1367) in the occurrence of TAs among the 108 cases of cryptorchidism (67 had appendages) and 30 fetal testicles (23 had appendages). When analyzing the position of the testes and the occurrence of appendices we found a significant difference between the inguinal testicles and fetuses (*p* = 0.0426), between the abdominal and suprascrotal testes (*p* = 0.0170), and between the inguinal and suprascrotal testes (*p* = 0.0022). No differences were found in the occurrence of appendices between the abdominal testicles and fetuses (*p* = 0.3904); between suprascrotal and fetal (*p* = 0.0570); and between abdominal and inguinal (*p* = 0.5425).

Of the 67 testicles with appendages, 49 (73.13%) had patent PV and 19 (28.35%) had EAs, whereas in 41 testicles without appendages, 23 (56.09%) had patent PV and 17 (41.46%) had EAs. This analysis showed that the presence of appendices is associated more frequently with patency of PV and with EAs (*p* = 0.0054). The correlation between PV patency, the occurrence of EAs, and the presence of TAs can be seen in Table 2.

## 4. Discussion

Knowledge of anomalies associated with cryptorchidism is relevant in clinical practice, both to prevent accidents during orchidopexy and to predict infertility in the future (and counsel patients/parents), such as in cases of epididymis atresia and total disjunction between the testis and the epididymis [19, 20]. EAs are frequently found in cryptorchidism [5, 13, 14]. The abdominal testicles present a higher index of these anomalies [3, 19]. Previous studies on fetuses and children without cryptorchidism have demonstrated an incidence of EAs below 4% [15, 16].

In the present study, we observed EAs in only 3.44% of the fetal testes and 36.12% in the cryptorchid testicles, a difference that was significant. When we analyzed the testicular position, we observed that the abdominal testicles presented EAs in half of the cases and those in the canal had EAs in more than 38%. The most cranially positioned testicles had a higher incidence of EAs than the suprascrotal ones and the fetal testicles located in the scrotum, confirming findings from previous studies [3, 5, 13].

Platt [18] questioned the high incidence of EAs associated with cryptorchidism [5, 7, 21]. The author considers this a consequence of the lack of definition of the normal anatomical pattern of the epididymis in the various studies. We used the same standard proposed by Turek [15] to analyze the relationship between the testis and the epididymis and found an incidence of more than 30% of this type of anomaly (disjunction and/or atresia) in patients with cryptorchidism, confirming the high incidence of EAs in cryptorchidism.

The timing of vaginal closure is still unknown. Studies suggest that at birth there would be patency of PV in up to 80% of boys, with progressively lower rates during the first year of life [22]. In a significant number of adult men, the PV is never obliterated. However, in the majority of these cases, there is no development of indirect inguinal hernia [5, 9]. In a study of 137 patients with cryptorchidism, the authors found no significant difference in the patency of PV in relation to the age of the patients [23].

The patency of PV in patients with cryptorchidism ranges from 21.3 to 81.3% [24]. In our study, PV was found in more than 66% of cases of cryptorchidism and 23% of fetuses, a significant difference. Regarding testicular position, we observed that all abdominal testicles had patent PV and that the testicles located in the canal presented a PV patency index of 64%, which was higher than the PV patency found in the suprascrotal testes and fetal testes.

Patients with cryptorchidism with patent PV have a higher EA index than in cases where the PV is closed [5, 7, 13]. The index of EAs in patients with cryptorchidism and patent PV varies from 50 to 80% [5, 8]. Of the 72 testicles with patent PV in our study, 27 (37.5%) had EAs and 12 (33.33%) had EAs in the 36 testicles that had occluded PV, a difference that was not significant. This finding, discordant with several studies in the literature, can be attributed to the type of classification used to determine epididymal anomalies. In our study we used the classification that is currently accepted in the literature [2, 8, 15]. However, when analyzing and comparing cases of EAs in fetuses and patients (72.5% of PV patency) with cases of normal epididymis anatomy in the two groups (53.12% of PV patency), the difference was significant, which confirms the association between EAs and PV patency.

TAs had a significantly lower incidence in patients with cryptorchidism, which could indicate a possible role of TAs in the process of testicular migration [12]. In our study, we observed that 62% of testicles with cryptorchidism had testicular appendages, a much larger number than the 24% reported by Józsa [12], who in an elegant histologic studied analyzed 37 appendix testes that were collected intraoperatively. The great majority of the population analyzed by Józsa was Caucasian. In contrast, we analyzed more than 100 testes of patients with a great variety of ethnicities. The substantial difference between our findings and those of Józsa might be explained by geographical or racial causes. There may be some differences in testicular appendix incidence in the different ethnicities. More prospective studies are needed to confirm this hypothesis.

We did not find a significant difference in the number of appendages in the testicles with cryptorchidism in relation to the control group, nor did we find a significant difference in the incidence of appendices in relation to testicular position in patients with cryptorchidism. Tostes [25] also did not observe differences in the number of TAs in the testicles of patients with cryptorchidism in relation the control group. This study showed that the TAs in undescended testes had a larger quantity of elastic fibers and smaller quantity of smooth muscle cells and predominance of type III collagen. The collagen matrix at the testicular appendix in patients with cryptorchidism is disrupted or degraded, rather than fibrotic, which is consistent with higher hydrostatic pressure. This finding suggests that undescended testis involves histologic alteration in TAs [25].

In the testes where the TAs were present, 73% had patent PV and 28% EAs. When comparing the incidence of EAs and the patency of PV in appendage patients with patients without appendices, we observed that the presence of appendices was associated more frequently with the patency of PV and EAs. The novelty of this report is the investigation of testicular appendages among the previously studied parameters, such as the position of the cryptorchidic testis, the presence of the abnormalities of the epididymis, and patency of the vaginal process.

The main limitations of our study are as follows: (a) the ideal control group for our study would consist of children with the same age group as those with cryptorchidism and without genital anomalies, a very difficult group to obtain, and (b) use of human fetuses as a control group. How can one know if the testicles will not become ascending (a previously documented scrotal testicle that later ascends into an extrascrotal position)? We do not have this answer, but the low PV patency index, lower than previously described in the literature [22], in our opinion may be a factor that demonstrates that the testicles present a lower risk of ascending.

## 5. Conclusions

The abdominal and inguinal testes were associated with the occurrence of a greater number of anatomical abnormalities of the epididymis and in the patency of the PV. The PV patency was not associated with a higher frequency of epididymal anomalies. The presence of paratesticular appendages was associated more frequently with the occurrence of epididymal anomalies and the patency of the vaginal process in patients with cryptorchidism.

## Figures and Tables

**Table 1:** The table shows the relation between the presence of testicular appendix, the patency of the vaginal process, and the presence of epididymal anomaly in patients with cryptorchidism in relation to the position of the testis.

*Testicular position*	*Appendices* presence/absence	*Processus vaginalis* occlusion/patency	*Epididymal anomalies* presence/absence
Abdominal (14—12.96%)	9 (64.29%)/5 (33.71%)	0 (0%)/14 (100%)	7 (50%)/7 (50%)
Inguinal (81—75%)	45 (55.66%)/36 (44.34%)	29 (35.8%)/52 (64.2%)	31 (38.27%)/50 (61.72%)
Suprascrotal (13—12.03%)	13 (100%)/0 (0%)	7 (53.84%)/6 (46.16%)	1 (7.69%)/12 (92.30%)

Total (108—100%)	67 (62.03%)/41 (37.97%)	36 (33.34%)/72 (66.66%)	39 (36.12%)/69 (63.88%)

**Table 2:** The table shows the correlation between the patency of the process and the presence of anomalies of epididymis and testicular appendices in the 108 testicles of patients with cryptorchidism studied.

Processus vaginalis	Epididymal anomalies	Testicular appendices
Patency: 72 (66.66%)	27 (69.23%)	49 (73.13%)
Occlusion: 36 (33.33%)	12 (30.76%)	18 (26.86%)

*Total: 108 (100%)*	*39 (100%)*	*67 (100%)*

## References

[1] Heyns C. F., Hutson J. M. (1995). Historical review of theories on testicular descent. *The Journal of Urology*.

[2] Sampaio F. J. B., Favorito L. A. (1998). Analysis of testicular migration during the fetal period in humans. *The Journal of Urology*.

[3] Lee P. A., Houk C. P. (2013). Cryptorchidism. *Current Opinion in Endocrinology, Diabetes and Obesity*.

[4] Hutson J. M., Thorup J. (2015). Evaluation and management of the infant with cryptorchidism. *Current Opinion in Pediatrics*.

[5] Scorer C. G., Farrington G. H. (1971). *Congenital Deformities of the Testis and Epididymis*.

[6] Barthold J. S., Redman J. F. (1996). Association of epididymal anomalies with patent processus vaginalis in hernia, hydrocele and cryptorchidism. *The Journal of Urology*.

[7] Mollaeian M., Mehrabi V., Elahi B. (1994). Significance of epididymal and ductal anomalies associated with undescended testis: study in 652 cases. *Urology*.

[8] Favorito L. A., Costa W. S., Sampaio F. J. B. (2006). Analysis of anomalies of the epididymis and processus vaginalis in human fetuses and in patients with cryptorchidism treated and untreated with human chorionic gonadotrophin. *BJU International*.

[9] Johansen T. E. B. (1987). Anatomy of the testis and epididymis in cryptorchidism. *Andrologia*.

[10] Lao O. B., Fitzgibbons R. J., Cusick R. A. (2012). Pediatric inguinal hernias, hydroceles, and undescended testicles. *Surgical Clinics of North America*.

[11] Rolnick D., Kawanoue S., Szanto P., Bush I. M. (1968). Anatomical incidence of testicular appendages. *The Journal of Urology*.

[12] Józsa T., Dienes B., Telek A., Hargitai Z., Pór Á., Kiss C. (2008). Differential expression of androgen and estrogen receptor of appendix testis in patients with descended and undescended testes. *International Journal of Urology*.

[13] Kim S.-O., Na S. W., Yu H. S., Kwon D. (2015). Epididymal anomalies in boys with undescended testis or hydrocele: Significance of testicular location. *BMC Urology*.

[14] Sharma S., Sen A. (2013). Complete testicular epididymal dissociation in the abdominal cryptorchid testis. *Journal of Pediatric Urology*.

[15] Turek P. J., Ewalt D. H., Snyder H. M., Duckett J. W. (1994). Normal epididymal anatomy in boys. *The Journal of Urology*.

[16] Favorito L. A., Sampaio F. J. B. (1998). Anatomical relationships between testis and epididymis during the fetal period in humans (10-36 weeks postconception). *European Urology*.

[17] Hern W. M. (1984). Correlation of fetal age and measuraments between 10 and 26 weeks of gestation. *Obst Gynecol*.

[18] Platt L. D., Medearis A. L., DeVore G. R., Horenstein J. M., Carlson D. E., Brar H. S. (1988). Fetal foot length: relationship to menstrual age and fetal measurements in the second trimester. *Obstetrics & Gynecology*.

[19] Han C. H., Kang S. H. (2002). Epididymal anomalies associated with patent processus vaginalis in hydrocele and cryptorchidism. *Journal of Korean Medical Science*.

[20] Rachmani E., Zachariou Z., Snyder H., Hadziselimovic F. (2012). Complete testis-epididymis nonfusion anomaly: a typical association with cryptorchid testis. *Urologia Internationalis*.

[21] Kubota M., Nakaya K., Arai Y., Ohyama T., Yokota N., Nagai Y. (2014). The area and attachment abnormalities of the gubernaculum in patients with undescended testes in comparison with those with retractile testes. *Pediatric Surgery International*.

[22] Burgmeier C., Dreyhaupt J., Schier F. (2014). Comparison of inguinal hernia and asymptomatic patent processus vaginalis in term and preterm infants. *Journal of Pediatric Surgery*.

[23] Favorito L. A., Costa W. S., Sampaio F. J. (2005). Relationship between the persistence of the processus vaginalis and age in patients with cryptorchidism. *International Brazilian Journal of Urology*.

[24] Aggarwal H., Kogan B. A., Feustel P. J. (2012). One third of patients with a unilateral palpable undescended testis have a contralateral patent processus. *Journal of Pediatric Surgery*.

[25] Tostes G. D., Costa S. F., Carvalho J. P. D., Costa W. S., Sampaio F. J. B., Favorito L. A. (2013). Structural analysis of testicular appendices in patients with cryptorchidism. *International Brazilian Journal of Urology*.

